# A retrospective comparative study of robot-assisted unilateral biportal endoscopic lumbar decompression and fusion surgery versus percutaneous endoscopic lumbar decompression and fusion surgery

**DOI:** 10.1097/MD.0000000000039664

**Published:** 2024-09-27

**Authors:** Yan dong Liu, Qiang Deng, Li xia Han, Kai dong Zhang, Yan jun Zhang, Ran dong Peng, Hai yun Yang, Tie feng Guo, Jun jie Li, Bo Chen, Sheng Tan

**Affiliations:** a Graduate School of Gansu University of Traditional Chinese Medicine, Lanzhou, Gansu, China; b Spinal Diseases Diagnosis and Treatment Center of Gansu Provincial Hospital of Traditional Chinese Medicine, Lanzhou, Gansu, China; c Lanzhou Orthopedic Hospital of Traditional Chinese Medicine, Lanzhou, Gansu, China; d Osteomyelitis Department of Gansu Provincial Hospital of Traditional Chinese Medicine, Lanzhou, Gansu, China.

**Keywords:** clinical research, minimally invasive spinal surgery, robot, unilateral biportal endoscope, vertebral decompression fusion

## Abstract

The objective of this study is to illustrate the advantages of robot-assisted unilateral biportal endoscopy in lumbar decompression fusion and internal fixation surgery. According to the different surgical methods, we divided the 26 patients into 2 groups, robot-assisted unilateral biportal endoscopy for lumbar interbody fusion (R-ULIF) group and percutaneous endoscopic lumbar decompression and interbody fusion (Endo-LIF) group, with a 1:1 ratio. Gender, disease course, lesion site, fluoroscopy times, operative time, blood loss, postoperative hospital stay, screw placement success rate, fusion rate, complications rate, postoperative pain visual analog scale (VAS) (The VAS score is used only to evaluate pain in the lower back and legs.) Oswestry Disability Index (ODI) (The ODI score can serve as a reference indicator for evaluating the effectiveness of treatment for patients with low back pain, and has good responsiveness in assessing patients with chronic low back pain), and MacNab (The MacNab standard is divided into 4 levels: excellent, good, fair, and poor, which can be used to evaluate the therapeutic efficacy of certain spinal surgeries) standard efficacy evaluation were analyzed and compared between the 2 groups. All patients successfully completed the surgery. Compared with the Endo-LIF group, the R-ULIF group had fewer fluoroscopy procedures, less intraoperative blood loss, and shorter postoperative hospital stay (*P* < .05). The VAS scores and ODI scores of both groups significantly decreased at all-time points (*P* < .05). The ODI scores of the R-ULIF group were better than the Endo-LIF group at 1 month and 3 months after surgery (*P* = .017/*P* = .047), but there was no statistically significant difference between the groups before surgery and 1 week after surgery (*P* > .05). The efficacy was evaluated using the MacNab criteria at 6 months after surgery. The R-ULIF group has an excellent and good rate of 84.6%, while the Endo-LIF group has an excellent and good rate of 76.9% (*P* = 1.000). Robot-assisted unilateral biportal endoscopy for lumbar interbody and fusion surgery has shown short-term clinical efficacy in the treatment of lumbar disc herniation combined with lumbar instability, surpassing endoscopic lumbar interbody fusion surgery. Robot-assisted unilateral biportal endoscopy for lumbar interbody and fusion surgery has demonstrated high success rate in screw placement, minimal radiation exposure, less intraoperative blood loss, shorter hospital stay, and thus deserves further clinical promotion.

## 1 . Introduction

Lumbar disc herniation (LDH) refers to a syndrome characterized by the protrusion of the nucleus pulposus, which compresses the dura sac or spinal nerve roots, leading to symptoms such as lower back pain, sciatica, or radicular pain. It is the most common degenerative spinal disease and is often accompanied by lumbar instability. Decompression and fusion with internal fixation have long been considered the gold standard for treating LDH and lumbar instability.^[1]^ However, the large trauma and long recovery time associated with open spinal surgery have been a challenge for spine surgeons. With the development of the minimally invasive concept and the optimization of endoscopic instruments, minimally invasive decompression and fusion surgery is gradually replacing traditional open decompression and fusion surgery due to its advantages of rapid recovery and minimal trauma to the surrounding tissues around the incision. Unilateral biportal endoscopy (UBE) is an emerging endoscopic technique based on the concept of minimally invasive surgery. Unlike the currently mature spine endoscopy, UBE requires the creation of 2 portals on the same side, namely the observation portal and the working portal.^[2]^ The dual-channel design of UBE allows for free movement of the surgical field and dynamic handling of instruments, enabling accurate and minimally invasive performance of endoscopic decompression and endoscopic lumbar interbody fusion (Unilateral Biportal Endoscopy-LIF, U-LIF). The precise insertion of pedicle screws is an important supplement to interbody fusion surgery. It is a crucial step in restoring spinal biomechanical stability and ensuring the safety of internal fixation. Manual placement of screws under fluoroscopic guidance cannot meet the requirements for accuracy and safety. With the successful application of artificial intelligence-assisted and computer-assisted navigation technology in minimally invasive spinal surgery, robot-assisted systems have created favorable conditions for the accuracy and safety of screw placement.^[3]^ The combination of robot-assisted unilateral biportal endoscopy and lumbar decompression fusion with internal fixation combines the intelligence and precision of the robot with the flexibility and minimally invasive nature of UBE, achieving more advanced surgical techniques for optimal treatment outcomes and maximizing patient benefits. Although robot-assisted screw placement and UBE fusion technology have attracted widespread attention, there is still a lack of clinical efficacy comparisons between the 2 and other endoscopic minimally invasive procedures. This report retrospectively analyzes and compares the recent clinical efficacy and differences between 26 patients with LDH and lumbar instability who were treated with robot-assisted unilateral biportal endoscopy for lumbar interbody fusion (R-ULIF) and percutaneous endoscopic lumbar decompression and interbody fusion (Endo-LIF) at the Spinal Disease Diagnosis and Treatment Center of Gansu Provincial Hospital of Traditional Chinese Medicine from February 2021 to June 2022 to evaluate the advantages of R-ULIF. The results are as follows.

## 2. Data and methods

### 2.1 . Research object

A controlled study was conducted at the LDH Combined Vertebral Instability Treatment Center of Gansu Provincial Hospital of Traditional Chinese Medicine from February 2021 to September 2022.

Inclusion criteria were as follows: ① The diagnosis is confirmed by the radiologist who validates the imaging data, indicating LDH combined with single-level lumbar instability; ② accompanied by symptoms such as radiating pain and numbness in the lower back and legs; ③ duration of the disease exceeding 8 weeks with no improvement after more than 3 months of systematic conservative treatment; ④ follow-up for more than 3 months with complete clinical data; ⑤ age restriction is between 18 and 80 years old; ⑥ no history of spine surgery; ⑦ LDH is limited to 1 segment, with an accompanying unstable vertebral body (lumbar 1-sacral 1).

Exclusion criteria were as follows: ① patients with language impairments who are unable to complete follow-up visits smoothly; ② patients with multi-segmental lumbar vertebral instability (>2 segments); ③ patients with spinal tuberculosis, infection, tumor, or other diseases; ④ patients with past history of cardiac, pulmonary, hepatic, or renal insufficiency. This study was approved by the Medical Ethics Committee of Gansu Provincial Hospital of Traditional Chinese Medicine (No: 2021-020-01).

### 2.2 . General information

Please refer Table 1 for the statistical comparison of general data such as gender, age, lesion site, and disease duration for 2 groups of patients.

**Table 1:** Comparison of general information between 2 groups (*x̄* ± s).

Group	Age (years)	Gender		Course of disease (month)	Lesion site			
		Male	Female		Lumbar 2, 3	Lumbar 3, 4	Lumbar 4, 5	Lumbar 5, sacral 1
R-ULIF group (n = 13)	53.92 ± 9.86	6	7	33.0 (6.1, 81.0)	1	1	8	3
Endo-LIF group (n = 13)	52.38 ± 10.89	5	8	48.0 (6.0, 120.0)	1	2	7	3
t/χ^2^/Z value	0.378	0.158		‐0.385	0.400			
*P* value	.709	1.000		.700	.940			

### 2.3. Operation method

R-ULIF group: with unstable lumbar 4. Using lumbar _4–5_ disc herniation as an example.

**Figure 1.**
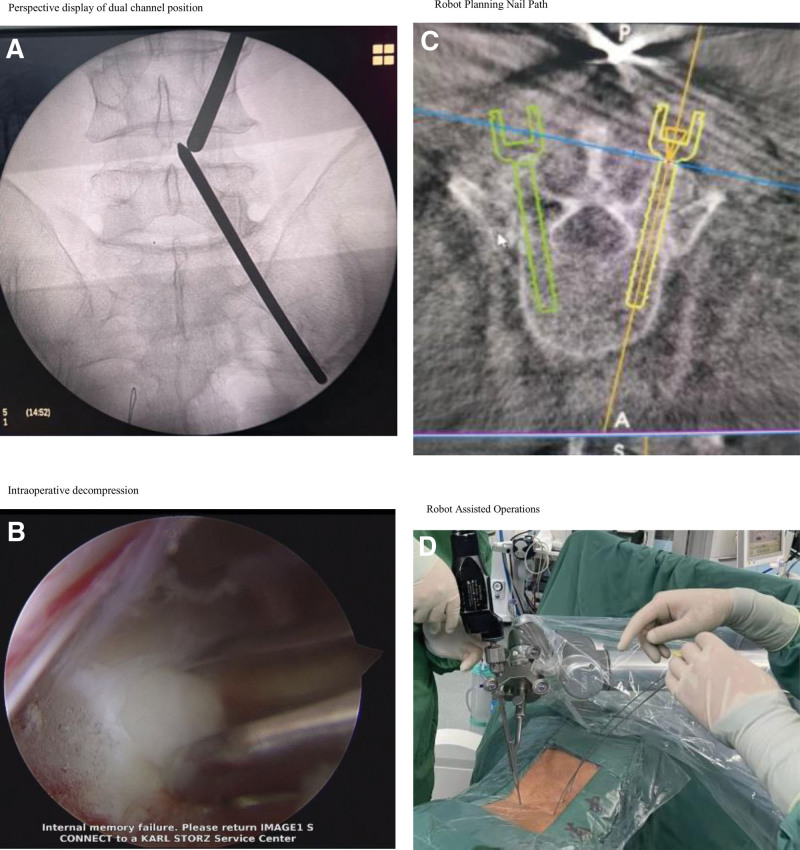
R-ULIF surgical operation diagram. (A) Perspective display of dual-channel position. (B) Intraoperative decompression. (C) Robot planning nail path. (D) Robot-assisted operations.

**Figure 2.**
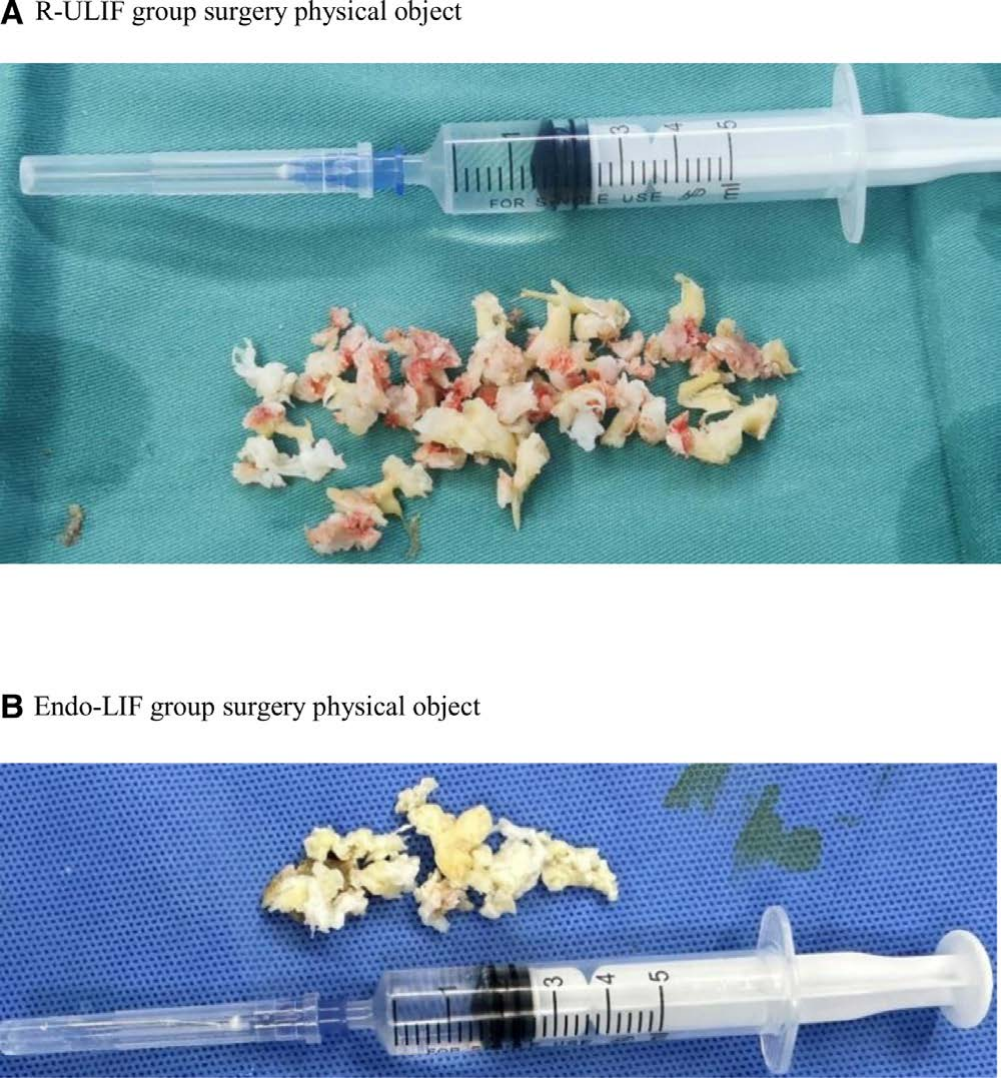
Two sets of physical illustrations of surgeries. (A) R-ULIF group surgery physical object. (B) Endo-LIF group surgery physical object.

1. Localization: the patient is placed in a prone position and a silicone prone support is placed on the abdomen. The left lumbar 4/5 space is located under 3D C-arm fluoroscopy (SIEMENS, Germany), and a horizontal line is marked on the surface of the body under fluoroscopy. A vertical line is marked 1 cm outside the center of the left lumbar 4 and lumbar 5 pedicle on the horizontal line, and a transverse incision line is marked 1.5 cm above the intersection of the horizontal and vertical lines. The surgical area is disinfected and sterile drapes are applied.
2. Decompression: approximately 1 cm incisions are made at the 2 marked incision sites, and progressive soft tissue dilators are inserted through the incisions towards the lumbar 4/5 lamina under fluoroscopic confirmation that the dilator tip is located at the root of the lumbar 4/5 spinous process. The fluoroscopy imaging system is adjusted to clear images and white balance is adjusted. An endoscope is inserted through the observation channel incision and a low-temperature plasma knife is inserted through the operating channel incision. Hemostasis and ablation of tissue are performed while continuously rinsing with saline. The surface of the yellow ligament is exposed, and the lumbar 4/5 lamina and yellow ligament between the lamina are alternately bitten open using a bur and a lamina rongeur, gradually expanding the laminar window and lateral recess to expose the nerve root and dura mater. Adhesions between the dura mater and extradural tissue are observed and separated using a nerve hook. The nerve hook is inserted into the spinal canal, pulling the lumbar 5 nerve root and dura mater towards the midline, and the protruding nucleus pulposus enters the field of view. The protruding and loose nucleus pulposus tissue is removed by using the nerve hook to pull the dura mater and nerve root, and the annulus fibrosus is incised with a sharp knife for intervertebral nucleus treatment.
3. Endplate preparation and bone fusion: the intervertebral space is expanded using a disc distractor, and the lumbar _4–5_ disc space is gradually prepared using 7 to 12 mm intervertebral curettes. The cartilage on the endplates of the vertebral bodies is scraped off using an intervertebral scraper. A fusion cage is trial-fitted, and after removing the trial-fitting, 5 mL of autogenous bone + allogeneic bone mixed particles are implanted into the disc space through a bone graft funnel. A 10 × 22 mm PEEK fusion cage (Kanghui Medical Equipment Co., Ltd., China) is inserted into the disc space. During the operation, fluoroscopy confirms the position of the fusion cage. Then the endoscope is inserted again to clean up any remaining bone fragments in the spinal canal. The intact dura mater and relaxed nerve roots are discovered during exploration. There is no active bleeding observed under the microscope, so the endoscope and working channel are removed.
4. Screw placement: the target vertebral body is located under C-arm fluoroscopy and marked on the surface of the body. Routine disinfection is performed, and sterile drapes are applied. The robot tracker and ruler are installed. CT three-dimensional reconstruction is performed with lumbar 4 as the center. The patient’s CT data is imported into the robot. The needle path for lumbar 4 and lumbar 5 is planned. After successful planning, the guide wires are placed through the lumbar 4 and lumbar 5 pedicles with the guidance of the robot. After confirming the ideal position of the guide wires under fluoroscopy, the pre-measured pedicle screws (6.5 × 45 mm for lumbar 4, 6.5 × 45 mm for lumbar 5) are successively screwed in along the guide wires. The ideal position of the pedicle screws is confirmed under fluoroscopy. A 60 mm connecting rod is installed on both sides of the pedicle screws, and the lumbar 5 screw cap is fixed. The vertebral bodies are reduced by distraction and the screw caps are tightened. Fluoroscopy again confirms that the pedicle screws are located within the corresponding vertebral bodies and pedicles, the position of the fusion cage is satisfactory, and lumbar 4 has been well reduced. The incision is flushed and the wound is thoroughly hemostatic. 10 mL of 0.375% ropivacaine hydrochloride is injected subcutaneously, a negative pressure drainage tube is placed and fixed, and the deep fascia, subcutaneous tissue, and skin are sutured layer by layer. The wound is dressed with sterile gauze. (See Fig. 1 for operative diagram of R-ULIF procedure, and Fig. 2 for the actual operation).

Endo-LIF group: taking lumbar 4 instability and lumbar _4–5_ disc protrusion as an example.

1. Localization and marking: general anesthesia is administered. The patient is placed in prone position, and a silicone prone position support frame is used to suspend the abdomen to adequately separate the intervertebral space. Under the guidance of a 3D C-arm X-ray machine (SIEMENS, Germany), the lumbar _4–5_ intervertebral space is located and marked on the skin. The surgical area is disinfected and sterile drapes are placed.
2. Endoscope placement: using the C-arm machine as a guide, a puncture is made towards the lumbar _4–5_ intervertebral foramen. Anteroposterior fluoroscopy confirms that the distal tip of the puncture needle is in the plane of the lumbar _4–5_ intervertebral space, while lateral fluoroscopy confirms that the puncture needle is positioned at the posterior superior edge of the lumbar 5 vertebral body. After making a 0.9 cm incision at the puncture point, a guidewire is inserted through the needle, and then a series of tissue dilators and working channels (1–4 levels) are inserted along the guidewire. C-arm fluoroscopy confirms that the front end of the channel is located at the ventral aspect of the lumbar 5 anterior articular base. The tissue dilators are removed, and the major access endoscope (iLESSYS Delta scope) is connected. The mirror system is adjusted until the image is clear, and continuous flushing with saline is performed.
3. Decompression procedure: under endoscopic visualization, the facet ring is trimmed and the ventral part of the lumbar 5 anterior articular base is removed, while preserving the articular surface. The lateral yellow ligament is removed to expose the nerve root. By probing the ventral side of the nerve root, free nucleus pulposus is observed and removed. The exploration continues towards the ventral, cranial, and caudal sides of the nerve root, and the protruding disc is removed. The decompressed nerve root is examined and found to be intact and relaxed. Bipolar radiofrequency is used for hemostasis and ablation of the annulus fibrosus.
4. Endplate preparation and bone graft fusion: a “U”-shaped retractor is used to protect the nerve root. The disc space is prepared by scraping the cartilaginous endplate, and a spot bleeding bone surface is preferred. A fusion cage is trial-fitted, and after removing the trial-fitting, 5 mL of autogenous bone + allogeneic bone mixed particles are implanted into the disc space through a bone graft funnel. A 10 × 22 mm PEEK fusion cage (Kanghui Medical Equipment Co., Ltd., China) is inserted into the disc space. Explore the nerve root again and find that the nerve root relaxation is ideal. Under endoscopic observation, there is no active bleeding, and the endoscope and working channel are removed.
5. Screw implantation: two percutaneous pedicle screws are implanted on each side of the lumbar 4 and lumbar 5 bodies. After confirming the satisfactory position of the pedicle screws under fluoroscopy, a percutaneous rod is installed to connect them. The lumbar 4 body is reduced and the screws are tightened for fixation. X-rays show that the pedicle screws are positioned correctly within the corresponding vertebral bodies and pedicles, and the fusion cages are placed satisfactorily. The surgical incision is washed with sterile saline and closed layer by layer, followed by sterile dressing.

### 2.4 . Postoperative treatment

Twenty hours after surgery, enoxaparin sodium injection was administered to prevent deep vein thrombosis; mannitol injection and dexamethasone sodium phosphate injection were given to alleviate postoperative edema; ceftriaxone sodium was used for anti-inflammatory and anti-infective purposes. Bedside functional exercises were conducted under the guidance of a physician on the first day after surgery, and ambulation activities began on the second day after surgery. Within 3 months postoperatively, wearing orthotics and gradually strengthening the muscles of the back and waist were required.

### 2.5 . Evaluation indicators

Record and compare general information of 2 groups of patients, including number of radiographs, operation time, blood loss, postoperative hospital stay, complication rate, VAS score at 3 days postoperatively, and Oswestry Dysfunction Index (ODI index); follow-up imaging data to assess screw fixation quality based on Gertzbein–Robbins classification criteria; follow-up at the 6th month based on imaging data and assess fusion rate according to Birdwell criteria. Evaluate clinical efficacy at 6 months postoperatively using modified MacNab criteria.

### 2.6 . Statistical methods

Data processing was conducted using the software SPSS 25.0. Normality test was performed for continuous variables, and for data that followed a normal distribution, they were presented as mean ± standard deviation ($\bar{x}$ ± s). For data that did not pass the normality test, the median (interquartile range) (M, X25%–X75%) was used. Paired *t* test was used for within-group comparisons, and independent samples *t* test was used for between-group comparisons. Repeated measures ANOVA was used for comparisons among multiple time points in 2 groups. If the sphericity assumption was violated, Greenhouse–Geisser correction was applied. Bonferroni correction was used for comparisons across different time points within the same group, and multifactorial ANOVA was used for comparisons across different groups at the same time point. Chi-square test or Fisher exact test was used for categorical data. Chi-square test was used for between-group comparisons. Chi-square test was used for between-group comparisons for ordinal data. The significance level was set at α = 0.05.

## 3. Results

### 3.1. Comparison of 2 groups of general data

A total of 26 eligible patients were included, with 13 in the R-ULIF group and 13 in the Endo-LIF group. There was no statistically significant difference in gender between the R-ULIF group and the Endo-LIF group (*P* = 1.000). The age of patients in the R-ULIF group (53.92 ± 9.86 years) and the Endo-LIF group (52.38 ± 10.89 years) showed no statistically significant difference (*P* = .709). The duration of illness in the R-ULIF group (33.0 [6.1, 81.0]) and the Endo-LIF group (48.0 [6.0, 120.0]) also showed no statistically significant difference (*P* = .700). There was no statistically significant difference in the affected lesion site between the R-ULIF group and the Endo-LIF group (*P* = .940) (see Table 1).

### 3.2. Comparison of the number of fluoroscopies, operative time, bleeding, postoperative hospital stay, nail placement accuracy, fusion rate, complication rate, and MacNab efficacy evaluation between the 2 groups

**Figure 3.**
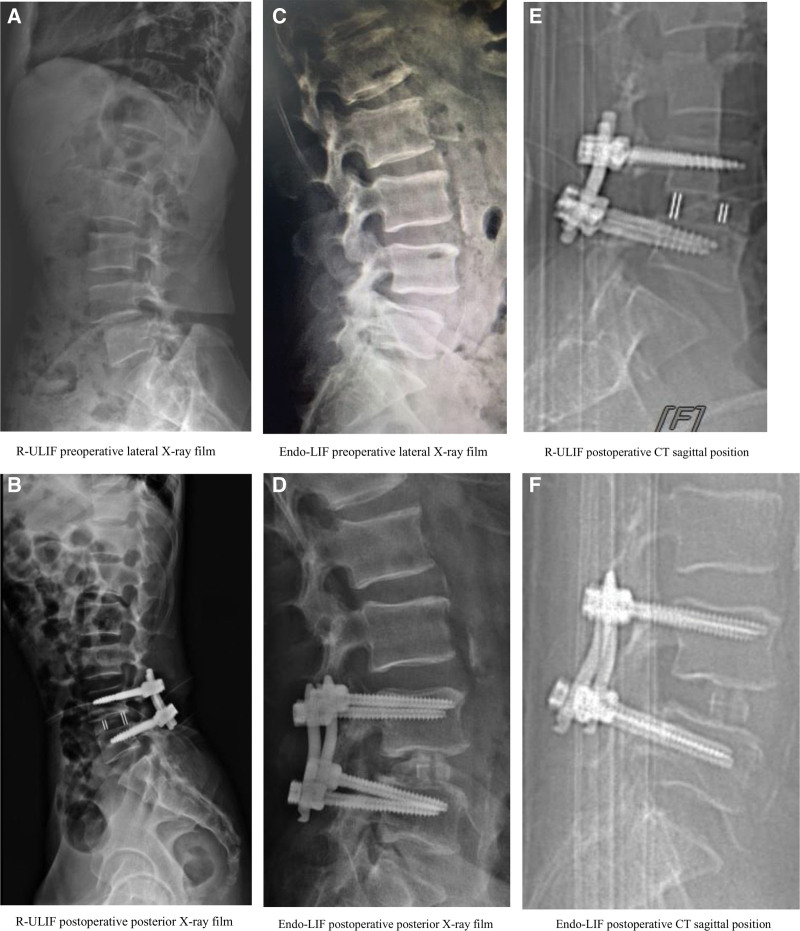
Comparison of imaging data before, after, and between the 2 groups. (A) R-ULIF preoperative lateral X-ray film. (B) R-ULIF postoperative posterior X-ray film; (C) Endo-LIF preoperative lateral X-ray film. (D) Endo-LIF postoperative posterior X-ray film; (E) R-ULIF postoperative CT sagittal position. (F) Endo-LIF postoperative CT sagittal position.

The number of fluoroscopic perspectives in the R-ULIF group (7.92 ± 0.95) was lower than in the Endo-LIF group (13.77 ± 1.48), and the difference was statistically significant (*P* < .05). The amount of bleeding in the R-ULIF group (54.54 ± 4.72 mL) was lower than in the Endo-LIF group (104.31 ± 6.81 mL), and the difference was statistically significant (*P* < .05). The postoperative hospital stay in the R-ULIF group (3.92 ± 0.86 days) was shorter than in the Endo-LIF group (5.23 ± 1.01 days), and the difference was statistically significant (*P* < .05). The operative time in the R-ULIF group (187.85 ± 10.18 min) was longer than in the Endo-LIF group (175.15 ± 23.81 min), but the difference was not statistically significant (*P* = .096) (see Table 2). The accuracy of nail placement in the R-ULIF group (98.1%) was higher than in the Endo-LIF group (76.92%), and the difference was statistically significant (*P* < .05). The fusion rate in the R-ULIF group (92.3%) was higher than in the Endo-LIF group (84.6%), but the difference was not statistically significant (*P* = 1.000). The incidence of complications in the R-ULIF group (0%) was lower than in the Endo-LIF group (15%), but the difference was not statistically significant (*P* = .593). The MacNab efficacy assessment in the R-ULIF group (84.6%) was higher than in the Endo-LIF group (76.9%), but the difference was not statistically significant (*P* = 1.000) (see Table 3). For a comparison of nail placement and fusion rates, please refer Figure 3.

**Table 2:** Comparison of relevant surgical indicators between 2 groups (*x̄* ± s).

Group	Operative time (min)	Number of perspectives (times)	Postoperative hospitalization time (days)	Intraoperative bleeding volume (mL)
R-ULIF group (n = 13)	187.85 ± 10.18	7.92 ± 0.95	3.92 ± 0.86	54.54 ± 4.72
Endo-LIF group (n = 13)	175.15 ± 23.81	13.77 ± 1.48	5.23 ± 1.01	104.31 ± 6.81
t/χ^2^ value	1.767	‐11.967	‐3.545	‐21.655
*P* value	.096	*P* < .001	.02	*P* < .001

**Table 3:** Comparison of clinical results between 2 groups (%).

Group	Nail placement accuracy (%)	Excellent rate of MacNab (%)	Fusion rate (%)	Incidence of complications (%)
R-ULIF group (n = 13)	98.1	84.6	92.3	0
Endo-LIF group (n = 13)	76.92	76.9	84.6	15
t/χ^2^ value	10.637	0.248	0.377	1.182
*P* value	.002	1.000	1.000	.593

### 3.3 . Comparison of visual analog score of postoperative pain (VAS) score and ODI score between the 2 groups

The results of this study showed that the group effect of VAS scores was not statistically significant between the 2 groups (F = 0.006, *P* = .937); the time effect of VAS scores was statistically significant between the 2 groups (F = 391.694, *P* < .001). The interaction between time and group was not statistically significant (F = 0.973, *P* = .410). There were no statistically significant differences in VAS scores between the 2 groups at preoperative, 1 week postoperative, 1 month postoperative, and 3 months postoperative time points (*P* > .05). Refer Table 4. The group effect of ODI scores was not statistically significant between the 2 groups (F = 3.935, *P* = .059). The time effect was statistically significant (F = 760.087, *P* < .001). The interaction between time and group was not statistically significant (F = 1.677, *P* = .180). Comparisons at different time points between the 2 groups showed no statistically significant differences in preoperative and 1 week postoperative ODI scores (*P* > .05), but the R-ULIF group had better ODI scores than the Endo-LIF group at 1 month postoperative (*P* = .017) and 3 months postoperative (*P* = .047). Refer Table 5.

**Table 4:** Comparison of VAS scores between 2 groups at different time points before and after surgery (*x̄* ± s).

Group	VAS scores			
	Preoperative	1 week after surgery	1 month after surgery	3 month after surgery
R-ULIF group (n = 13)	6.85 ± 0.90	3.92 ± 0.86	2.00 ± 0.58	0.77 ± 0.60
Endo-LIF group (n = 13)	6.62 ± 1.26	4.30 ± 1.03	2.00 ± 0.71	0.69 ± 0.63
Statistical value	Time effect F = 391.694, *P* < .001			
	Interact effect F = 0.973, *P* = .410			
	GROUP effect F = 0.006, *P* = .937			

**Table 5:** Comparison of ODI scores between 2 groups at different time points before and after surgery (*x̄* ± s).

Group	ODI scores			
	Preoperative	1 week after surgery	1 month after surgery	3 month after surgery
R-ULIF group (n = 13)	56.77 ± 3.27	48.77 ± 3.09	28.62 ± 3.15	19.77 ± 2.09
Endo-LIF group (n = 13)	59.69 ± 5.01	49.08 ± 2.99	32.15 ± 3.87	21.62 ± 2.40
Statistical value	Time effect F = 769.087, *P* < .001			
	Interact effect F = 1.677, *P* = .180			
	Group effect F = 3.935, *P* = .059			

## 4. Discussion

Although open lumbar interbody fusion (LIF) is considered the gold standard for treating certain spine disorders due to its ability to permanently decompress nerve structures and achieve lasting biomechanical fusion between vertebrae using different approaches and implants, its large incision and deep surgical wound pose iatrogenic risks such as blood loss, complications, and disruption of spinal and attachment biomechanical stability.^[4]^ In our study, we used the Endo-LIF technique to treat LDH combined with vertebral instability. Through this study, we found both advantages and disadvantages of the technique. Firstly, compared to traditional open surgery, Endo-LIF reduces the damage to surrounding muscles and soft tissues, thus reducing postoperative pain and recovery time. Secondly, the endoscope provides a clear, magnified view, which can reduce the risk of damage to surrounding nerves and blood vessels during surgery. Additionally, the smaller incision size lowers the risk of postoperative complications such as infection and bleeding. Although the coaxial single-channel percutaneous spinal endoscope can effectively relieve the pathology, its minimally invasive nature may sometimes result in incomplete removal of the lesion, leading to disease recurrence. Moreover, the Endo-LIF technique is technically challenging and requires high surgical skills and experience from the surgeon. Inexperienced surgeons may encounter issues such as longer operation time and increased complications.^[5,6]^ Among the 13 patients included in our study who underwent Endo-LIF, 1 male patient experienced dural tear, and 1 female patient experienced fusion device subsidence, resulting in a complication rate of 15%. We believe that the dural tear was due to severe adhesion between the ligamentum flavum and dura mater, as well as the narrow operating range of the single-channel endoscope; the fusion device subsidence was due to patient osteoporosis and improper placement of the device. Furthermore, due to the narrow surgical channel, Endo-LIF may not be able to fully address severe or multilevel lesions. Intraoperative bleeding is an issue that needs attention regardless of the surgical technique, but in endoscopic surgery, bleeding can affect the surgical field of view and increase the difficulty of the operation. In addition, this surgery requires special equipment and instruments, which can lead to higher costs.^[7]^

In our study, we also used ULIF to treat LDH with vertebral instability. ULIF establishes independent observation and working channels through 2 percutaneous incisions on the same side of the spine. We found that this dual-channel design provides surgeons with more flexible operations and a broader field of view compared to a single-channel endoscope. Additionally, ULIF allows surgeons to use familiar conventional surgical instruments for endoscopic operations, and the UBE endoscopic view is similar to that of open surgery, achieving integration of open surgery and endoscopic surgery. This has been referred to as “endoscopic open surgery” in the literature.^[8,9]^ We found that the independent visualization capability of ULIF not only addresses the difficulties in positioning, narrow field of view, and low working efficiency of Endo-LIF but also improves the limitations of extensive-channel techniques, which can cause substantial iatrogenic damage and blurry vision. For patients, ULIF only requires 4 small incisions of 1.5 cm each, which can significantly reduce damage to normal anatomical structures such as muscles, ligaments, and bones, and has advantages such as thorough decompression of the spinal canal, minimized trauma, reduced blood loss, quick recovery, fewer complications, and high fusion rates.^[10]^ As shown in our study results, the R-ULIF group had lower blood loss and shorter postoperative hospital stays compared to the Endo-LIF group, with statistically significant differences. However, there were no statistically significant differences between the R-ULIF and Endo-LIF groups in terms of fusion rate, complication rate, and MacNab efficacy evaluation. It is evident that the R-ULIF group had longer operation times than the Endo-LIF group, which may be due to the use of robot-assisted implantation, which prolongs the surgery. During ULIF, we believe that surgeons should focus on several key issues: (1) accurate positioning, (2) proficiency in using ultrasonic osteotomes, (3) maintaining a clear surgical field of view, (4) thorough decompression, and (5) firm fusion.

Pedicle screw fixation helps promote local bone fusion and is an important step for anatomical reconstruction of the vertebral position in LIF. Percutaneous screw placement has advantages such as minimal trauma, reduced blood loss, less pain, and faster recovery. However, before the introduction of robot navigation technology, percutaneous screw placement relied mainly on manual techniques, which not only could not guarantee the accuracy of screw placement but also carried the risk of complications such as nerve and vascular injury, cerebrospinal fluid leakage, and reoperation due to failed screw placement.^[11]^ Precision treatment has become one of the main development directions in spinal surgery in the 21st century, especially with technologies such as digital orthopedics representing a hot research topic in minimally invasive medicine.^[12]^ Robot navigation positioning systems can utilize their tracking capability and intraoperative three-dimensional navigation system for spatial positioning and path navigation, thereby improving the accuracy and safety of surgery. As a result, it is increasingly favored by orthopedic surgeons. The “Tianji” robot used in the R-ULIF group in this study is an intelligent navigation and positioning system based on three-dimensional imaging technology. The system features active positioning and human–machine cooperation, which achieves precise percutaneous screw implantation according to the preoperative planned path, thereby improving surgical efficacy.^[13]^ In this study, the accuracy rate of screw placement was 98.1%, far exceeding the 76.92% accuracy rate of manual placement; in terms of radiation exposure, the R-ULIF group had a lower number of fluoroscopy shots (7.92 ± 0.95) compared to the Endo-LIF group (13.77 ± 1.48), indicating significant advantages of robot-assisted screw placement in terms of accuracy, safety, and consistency. However, we should strive to improve the proficiency and skills in the application of robots and minimize the total surgical time. With the development of optical imaging technology and artificial intelligence, the decompression fusion method of LIF has evolved from Endo-LIF to UBE microscope-assisted decompression fusion, and the screw placement method has also evolved from manual placement to robot-guided placement. As patients’ demands for surgical quality continue to increase, the combination of these emerging minimally invasive techniques in practice becomes particularly important.

The technical characteristics of R-ULIF are the combination of the robot and ULIF technology, utilizing the UBE’s wide field of view and operating space to accomplish minimally invasive decompression fusion of the affected segment; and using the intelligence and precision of the surgical robot to perform percutaneous pedicle screw implantation. The advancement of R-ULIF lies in the synergy of the advantages of the 2 new technologies and the offsetting of their disadvantages. Under the surveillance of imaging equipment, the treatment objectives are achieved with the maximum minimally invasive and precision methods to maximize the benefits for patients. The perfect combination of doctors, robots, and spinal endoscopes represents a profound change from conventional open surgery with large incisions and deep wound cavities. This approach, which achieves the maximum effect with the minimal cost, aligns with the development concepts of minimally invasive surgery, intelligent surgery, and fast recovery surgery.^[14]^ However, as both robot-assisted technology and ULIF technology are still in their early stages, they have not yet fully matured or been widely adopted. From the longer surgical time in the R-ULIF group, it is evident that doctors’ proficiency and operating skills with robot assistance and ULIF technology still need further improvement. In addition, the current application range of robots in spinal endoscopic surgery is somewhat limited, as it mainly emphasizes precision while intelligence falls slightly short. In the future, we should further promote the perfect integration of robots and spinal endoscopes by expanding the functions of robots.

This study analyzed and compared the short-term clinical efficacy of R-ULIF and Endo-LIF in lumbar decompression fusion and internal fixation surgery. The limitations of this study include a small sample size and short follow-up period, which cannot obtain long-term clinical efficacy comparison results.

## Author contributions

**Conceptualization:** Qiang Deng.

**Data curation:** Kai dong Zhang.

**Formal analysis:** Ran dong Peng.

**Funding acquisition:** Qiang Deng.

**Investigation:** Yan jun Zhang, Sheng Tan.

**Methodology:** Hai yun Yang.

**Project administration:** Qiang Deng.

**Validation:** Bo Chen.

**Visualization:** Jun jie Li.

**Writing – original draft:** Yan dong Liu.

**Writing – review & editing:** Li xia Han, Tie feng Guo.
